# Green Fuels Production: Parameter Estimation of Fatty Acid Hydrodeoxygenation from Batch Data

**DOI:** 10.1021/acsomega.5c11040

**Published:** 2026-02-01

**Authors:** Carlos Eduardo Korn Senko, Sara Regina Osipi, Mauro Antonio da Silva Sá Ravagnani

**Affiliations:** Chemical Engineering Graduate Program, 42487 State University of Maringá, Maringá, PR CEP 87020-900, Brazil

## Abstract

Replacing fossil fuels with renewable alternatives has become increasingly critical. The hydrodeoxygenation of fatty acids presents a viable route for green fuel production, and understanding the kinetics of these reactions is crucial for evaluating their economic viability. The present paper focuses on estimating the kinetic parameters for the hydrodeoxygenation of stearic and palmitic acids to produce *n*-alkanes, considering two reaction schemes: RS1, a detailed theoretical pathway model, and RS2, a simplified kinetic representation and categorization of similar reactions into families with linear free energy relationships (LFERs). Aspen Plus v14 was used as the process simulation environment to fit the batch data, employing the generalized Langmuir–Hinshelwood–Hougen–Watson kinetic model for RS1, while RS2 was described using a power-law kinetic expression, both using Soave–Redlich–Kwong as the thermodynamic model. To minimize the error between simulated and experimental data, the sum of squared errors was used as the objective function with an algorithm that integrates particle swarm optimization and golden search. RS2 demonstrated superior performance in global error minimization (*R* ^2^ of 0.985) and parameter reliability compared to RS1 (*R* ^2^ of 0.981). Grouping reactions into families using the LFER method successfully achieved reliable results. This approach shows promise for estimating kinetic parameters of systems with complex feedstocks while reducing the number of variables. Using kinetic-based models instead of conversion-based models enhanced the optimization of green fuel production units.

## Graphical abstract

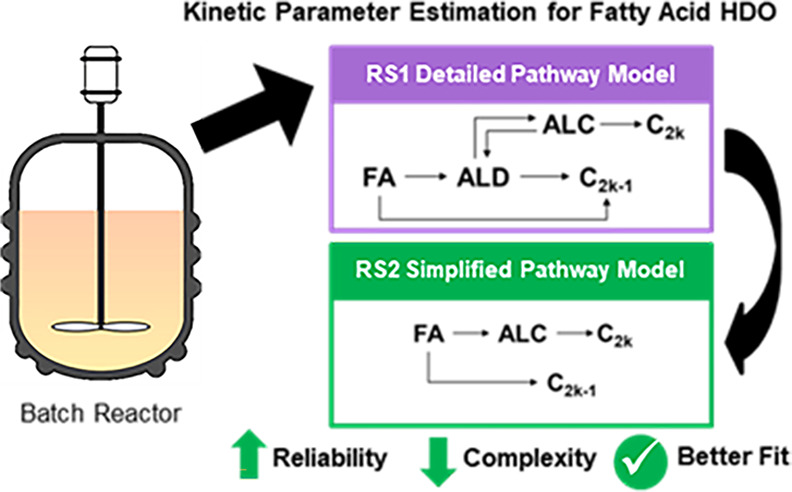

## 1 Introduction

Greenhouse gases (GHGs) emitted by human activities are a major contributor to climate change observed in recent decades. While fossil fuels remain a crucial energy source for modern society, they are also a primary source of GHG emissions. In contrast, renewable fuels offer a promising alternative to mitigate the environmental impact and reduce GHG emissions.

Renewable fuels are derived from natural resources that replenish themselves within relatively short periods. Examples include ethanol and biodiesel. However, some liquid fuels, such as diesel and jet fuel, have limitations on direct substitution with conventional renewable fuels, including freeze point restrictions and calorific value. Green fuels, developed as alternatives to these heavier liquid fuels, possess properties similar to those of their fossil fuel counterparts but are obtained from renewable materials. The hydroprocessed ether and fatty acid (HEFA) route is one of the most established technologies for producing green fuels. This process involves the catalytic hydrodeoxygenation (HDO) of esters and fatty acids derived from vegetable oils to produce alkanes.^1^ In recent years, numerous studies have focused on HDO, exploring aspects such as process simulation, parameter optimization, and economic or environmental impact assessments.^2–5^ However, many studies focus on product conversion rather than investigating the kinetics of the reactions involved. Understanding these kinetics is essential for properly sizing the reactors and utilities used in the process.

Estimating kinetic parameters normally requires the use of a simulator to replicate the experimental settings. Alongside this, nondeterministic optimization approaches, such as particle swarm optimization (PSO), play a critical role in minimizing the error between the experimental data and the simulated results. Estimating kinetic parameters for processes involving vegetable oils as feedstock often entails calculating a large number of variables due to the diverse fatty acids present. To simplify this complex modeling, a homologous series of reactants can be grouped into reaction families, each characterized by a unique set of parameters. This approach, described by the linear free energy relationships (LFERs) modeling strategy,^6^ enables the development of more parsimonious kinetic models, in which the conversion of fatty acids into products can be effectively described with fewer parameters, enhancing the model’s manageability.

In the present paper, the main objective was to estimate the kinetic parameters for the hydrodeoxygenation reactions of stearic acid and palmitic acid by applying the reaction family approach. As a secondary objective, this study evaluates the reliability and statistical significance of the estimated parameters through confidence interval analysis, allowing the identification of reaction pathways that most strongly influence the model accuracy.

The remainder of the paper is structured as follows: Section 2 presents the methodology, including the experimental data set, reaction schemes, kinetic formulation, simulation setup, LFER implementation, and optimization procedures, together with the algorithm description and confidence interval estimation. Section 3 presents and discusses the results obtained for RS1 and RS2, and Section 4 summarizes the main conclusions and outlines perspectives for future work.

## 2 Methodology

### 2.1 Data Set

Data input for this study was derived from Yenumala et al.^7^ In a 300 mL reactor, 5 g of triglycerides were fed in a 1:2 molar ratio of tripalmitin and tristearin, diluted with 100 mL of n-dodecane. The reactor was pressurized to 30 bar with hydrogen (H_2_), and experiments were conducted across temperatures ranging from 553 to 633 K. The catalyst employed consists of 15% by weight of nickel supported on γ-Al_2_O_3_, denoted as 15NiAl. Results obtained by Yenumala et al.^7^ are categorized into 166 data points, comprising: (1) stearic acid (SA) conversion with temperature variation; (2) palmitic acid (PA) conversion with temperature variation; (3) concentrations of products over reaction time at 613 K, categorized by fatty acid origin; and (4) temperature effects on product distribution. Yenumala et al.^7^ highlighted that chromatograms of the reactor outlet stream showed no presence of aldehydes. This justifies the use of zero aldehyde concentrations in groups (3) and (4), ensuring that the parameter estimation process reflects the experimentally observed absence of this intermediate.

### 2.2 Reaction Scheme

Throughout this work, two reaction schemes were simulated: reaction scheme 1 (RS1), which comprises five reactions (including one equilibrium), and reaction scheme 2 (RS2), a simplified version with three reactions. Both schemes are based on the conversion of fatty acids (e.g., stearic and palmitic acids) into *n*-alkanes through distinct pathways: hydrodeoxygenation (HDO), decarbonylation (DCn), and decarboxylation (DCx).

Based on Arora et al.,^8^ RS1, depicted in Figure 1, was modified to include the reversibility of the aldehyde-to-alcohol reaction, as proposed by Yenumala et al.^7^ Aldehyde-to-alcohol reversibility has been observed in another study using 15NiAl.^9^

**Figure 1:**
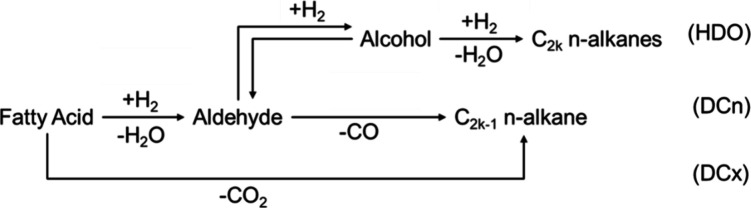
RS1 is for fatty acids. C_2*k*_ represents *n*-alkanes with an even number of carbons, while C_2*k*–1_ with an odd number.

To standardize notation, reaction paths are represented in this work by connecting reactants and products with an arrow in the format reagent → product. For example, the conversion of a fatty acid into the corresponding aldehyde is denoted as FA_2*k*_ → ALD_2*k*_. In this notation, FA, ALD, ALC, and C correspond to fatty acid, aldehyde, alcohol, and alkane families, respectively, while the subscript indicates the carbon number of the molecule (*k* = 8 for palmitic reactions and *k* = 9 for stearic reactions).

Triglycerides are promptly converted to fatty acids. These acids then undergo reactions through three distinct pathways: hydrodeoxygenation (HDO), encompassing FA_2*k*_ → ALD_2*k*_, ALD_2*k*_ → ALC_2*k*_, and ALC_2*k*_ → C_2*k*_, yielding even-numbered *n*-alkanes and water; decarbonylation (DCn), including FA_2*k*_ → ALD_2*k*_ and ALD_2*k*_ → C_2*k*–1_, generating odd-numbered *n*-alkanes and CO; and decarboxylation (DCx), primarily FA_2*k*_ → C_2*k*–1_, which produces odd-numbered *n*-alkanes and CO_2_, the only pathway that does not consume H_2_.

Incorporating the decarboxylation route into the reaction scheme proposed by Yenumala et al.^7^ significantly modifies the reaction parameters for HDO and DCn. Consequently, the values estimated by Yenumala et al.^7^ cannot be used as benchmarks or compared directly to the estimates in this study.

Based on the confidence interval analysis of RS1, it was observed that the model may be overspecified. Some reactions were found to have a negligible impact on the calculated error, suggesting that the model could be simplified without a significant loss of accuracy. Therefore, RS2 was proposed, comprising three main reactions: conversion of fatty acids to their corresponding alcohols (FA_2*k*_ → ALC_2*k*_); direct conversion of fatty acids to odd-numbered *n*-alkanes via DCx (FA_2*k*_ → C_2*k*–1_); conversion of alcohols to even-numbered *n*-alkanes (ALC_2*k*_ → C_2*k*_). In summary, RS2, presented in Figure 2, consolidates the fatty acid → aldehyde and aldehyde → alcohol conversions into a single step and does not include the decarbonylation pathway present in RS1.

**Figure 2:**
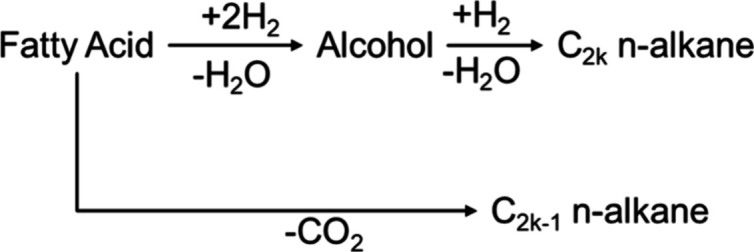
RS2 represents fatty acids. C_2*k*_ represents *n*-alkanes with an even number of carbons, while C_2*k*–1_ with an odd number.

### 2.3 Kinetics

The kinetic formulation for RS1 was developed based on the Langmuir–Hinshelwood (LH) approach proposed by Arora et al.,^8^ with the inclusion of the reversible aldehyde–alcohol step as suggested by Yenumala et al.^7^ According to Arora et al.,^8^ LH-type rate expressions capture competitive adsorption phenomena and inhibition effects caused by fatty acids on the catalytic surface. To derive the LH expressions, we assumed that fatty acids and hydrogen adsorb onto catalytically active sites, with the surface reaction acting as the rate-determining step, followed by product desorption. Since there are two fatty acids in Yenumala and collaborators’^7^ experimental data, both are considered for the adsorption constants. Table 1 presents reaction schemes and rate expressions considered in this work.

**Table 1:** Reaction Schemes and Rate Expressions for RS1

reaction scheme	rate expression
FA_2*k*_ + H_2_ → ALD_2*k*_ + H_2_O	$\frac{k_{1}C_{{\mathrm{FA}}_{2k}}C_{H_{2}}}{\left(1+\sum K_{\mathrm{FA}}C_{\mathrm{FA}}\right)}$
FA_2*k*_ → C_2*k*–1_ + CO_2_	$\frac{k_{2}C_{{\mathrm{FA}}_{2k}}}{\left(1+\sum K_{\mathrm{FA}}C_{\mathrm{FA}}\right)}$
ALD_2*k*_ + H_2_ → ALC_2*k*_	$\frac{k_{3}C_{{\mathrm{ALD}}_{2k}}C_{H_{2}}}{\left(1+\sum K_{\mathrm{FA}}C_{\mathrm{FA}}\right)}$
ALC_2*k*_ → ALD_2*k*_ + H_2_	$\frac{k_{-3}C_{{\mathrm{ALC}}_{2k}}}{\left(1+\sum K_{\mathrm{FA}}C_{\mathrm{FA}}\right)}$
ALD_2*k*_ → C_2*k*–1_ + CO	$\frac{k_{4}C_{{\mathrm{ALD}}_{2k}}}{\left(1+\sum K_{\mathrm{FA}}C_{\mathrm{FA}}\right)}$
ALC_2*k*_ + H_2_ → C_2*k*_ + H_2_O	$\frac{k_{5}C_{{\mathrm{ALC}}_{2k}}}{\left(1+\sum K_{\mathrm{FA}}C_{\mathrm{FA}}\right)}$

The second reaction scheme (RS2) was derived after evaluating the parameter confidence intervals obtained for RS1. As discussed in Section 3.2, the DCx route proved more statistically reliable than DCn, enabling removal of the decarbonylation pathway without loss of accuracy. Since aldehydes were not detected experimentally and the forward ALD_2*k*_ → ALC_2*k*_ step is significantly faster than the reverse, the intermediate and its reversibility were lumped into FA_2*k*_ → ALC_2*k*_. Additionally, adsorption constants approached negligible values, justifying the simplification from a Langmuir–Hinshelwood to a power-law formulation. The reactions and respective power-law rate formulations adopted in RS2 are summarized in Table 2.

**Table 2:** Reaction Schemes and Rate Expressions for RS2

reaction scheme	rate expression
FA_2k_ + H_2_ → ALC_2*k*_ + H_2_O	*k*_1_C_FA_2*k*__C_H_2__
FA_2*k*_ → C_2*k*–1_ + CO_2_	*k*_2_C_FA_2*k*__
ALC_2*k*_ + H_2_ → C_2*k*_ + H_2_O	*k*_5_C_ALC_2*k*__

### 2.4 Simulation

Aspen Plus v14 was employed as the process simulator, replicating the time-dependent experimental conditions. The *RBatch* reactor model was selected to emulate the experimental batch reactor configuration. The component mass balance for the liquid-phase batch reactor is given by

$$\frac{dC_{j}}{dt}=\sum_{i}\nu_{ij}r_{i}$$ 1

where *C*_*j*_ is the concentration of component *j*, *ν*_*ij*_ is the stoichiometric coefficient in reaction *i*, and *r*_*i*_ is the reaction rate defined in Section 2.3. All reactions were assumed to occur in the liquid phase, and reaction rates were expressed per unit mass of catalyst. As experiments were carried out under isothermal conditions and heat effects were considered negligible, no energy balance was included.

The reaction kinetics for RS1 were modeled using the generalized Langmuir–Hinshelwood–Hougen–Watson (GLHHW) mechanism, explicitly considering the selective adsorption of fatty acids onto the catalyst surface. In contrast, RS2 was described by using a power-law kinetic expression. It was also considered that the reaction rate exponent for the organic reactant was one, while the dependence on hydrogen was incorporated into the reaction parameters due to its excess. For thermodynamic modeling, the Soave–Redlich–Kwong (SRK) method was used due to its compatibility with the process conditions and components.^5,8^ The reacting medium consists mainly of heavy hydrocarbons at high pressures and temperatures, for which nonideal behavior is expected. Therefore, the Soave–Redlich–Kwong (SRK) equation of state with classical mixing rules was selected to represent phase equilibria. More advanced models can also be employed. However, SRK is widely adopted in the literature for HDO systems, and the required parameters for heavy components in alternative models are not fully available in Aspen.

### 2.5 Linear Free Energy Relationships (LFERs)

Linear free energy relationships (LFERs) correlate reaction rates or equilibrium constants with structural properties of the reactants, allowing for the prediction of how molecular variations influence reaction behavior. This is particularly advantageous with complex feeds, where components can be grouped in a family. These components within the family, having the same reaction path, can share kinetic parameters such as pre-exponential factor and activation energy. In order to account for the different values observed in reaction rates for components within a family, this model incorporates an alpha factor (α_*i*_). The alpha factor adjusts the kinetic parameters by considering the reaction enthalpy of each individual component.^6^

The reaction rate constant is described by the Arrhenius law, eq 2, and the activation energy was obtained using the Bell-Evans–Polanyi equation within a reaction family, as shown in eq 3.^6^

$$\ln\;k_{ij}=\ln\;A_{0}-\frac{E_{ij}}{RT}$$ 2

$$E_{ij}=E_{io}+\alpha_{i}\Delta H_{r,ij}$$ 3

In these equations, *A*_0_ is the pre-exponential factor, *E*_*io*_ is the activation energy of the reaction family, and α_*i*_ is the factor that multiplies the reaction enthalpy (Δ*H*_*r*, *ij*_) of each reaction. The subscripts “*i*” and “*j*” represent the reaction family and the species, respectively.

### 2.6 Optimization

The sum of squared errors (SSE) was employed as the objective function to quantify the discrepancy between the observed experimental data and the model predictions, thereby minimizing the cumulative squared differences and ensuring the optimal fit of the kinetic parameters to the data. This is mathematically represented in eq 4.

$$\mathrm{Error}=\sum (y_{\exp}-y_{\mathrm{sim}}{)}^{2}$$ 4

where *y*_exp_ and *y*_sim_ are the experimental and simulated mole fractions of the component in the outlet stream, respectively. The error is reported in the same units as the mass fractions used in the objective function (dimensionless). For RS1, the optimization problem involved determining kinetic parameters for six reactions across six reaction families. Three variables were estimated per reaction family: the pre-exponential factor (*A*_0_), the activation energy of the reaction family (*E*_*io*_), and the α_*i*_ factor from the linear free energy relationships (LFERs) approach. Additionally, two adsorption constants (*K*_ADS_) were incorporated to account for fatty acid adsorption on the catalyst surface. This resulted in a total of 20 variables to be optimized. The bound values for each parameter were set as follows: *A*_0_ ranged from 10^–8^ to 10^–1^ kmol/kg_cat_ s, *E*_*io*_ ranged from 0 to 210 kJ/mol, α_*i*_ ranged from 0 to 1, and *K*_ADS_ ranged from e^0^ to e^7^.

For RS2, the number of reactions was reduced from six to three, eliminating the decarbonylation (DCn) pathway and consolidating reactions. Consequently, the adsorption constants (*K*_ADS_), which were specific to RS1, were no longer applicable. This reduction led to a significant decrease in dimensionality, lowering the number of optimization variables to 9, consisting of three parameters (*A*_0_, *E*_*io*_, and α_*i*_) for each of the three reaction families. The bound values for each parameter were set as follows: *A*_0_ ranged from 10^–8^ to 10^2^ kmol/kg_cat_ s, *E*_*io*_ ranged from 0 to 300 kJ/mol, and α_*i*_ ranged from 0 to 1.

These bounds were set for *A*_0_, *E*_io_, and *K*_ADS_ based on values found in related articles,^5,7,8,11^ while α_*i*_ is limited by conceptual design.^6^ Regarding constraints, there is a mathematical possibility for the activation energy to become a negative number, depending on the parameter values in eq 3. To address this issue and ensure physically meaningful results, a penalty value was incorporated into the objective function for every instance where the activation energy turned out to be negative. This approach effectively discourages the optimizer from selecting parameter sets that would result in nonphysical activation energy values, thereby maintaining the integrity of the kinetic model.

Python was chosen to code the optimization algorithm. Its extensive libraries offer a wide range of tools for data manipulation and optimization algorithms. These libraries can also be adapted to suit specific cases, providing flexibility in tailoring the algorithm to meet specific research requirements.

Particle swarm optimization (PSO) was selected as the first optimization tool for this study. PSO’s approach makes it particularly adept at exploring complex, high-dimensional search spaces. Moreover, PSO is well-suited for scenarios where the function evaluations are computationally expensive, or the function itself is not explicitly defined (black-box functions). PSO aggregates all normalized variables, varying them within a search range. The chosen normalized range was from 0 to 7 for RS1 and 0 to 10 for RS2, with each variable’s conversion factor within the particle depending on the variable itself. Considering *x* as the variable value within the particle, the conversions are defined as follows: *A*_0_ = 10^*x*^ – 8; *E*_*io*_ = 30*x*; α_*i*_ = *x*/7 or *x*/10; and *K*_ADS_ = *e* ^*x*^. For PSO’s parameter tuning, population, inertia, cognitive, and social components were kept as standard values for *pyswarm*, Python’s library. So, the population was set as 100 and *w* = *c*_1_ = *c*_2_ = 0.5. With this set of parameters, 20 iterations were proven enough to concentrate the particle population to a minimum. It is important to note that, for complex multimodal objective functions, no optimization method can provide a formal guarantee of reaching the global optimum. In this context, PSO offers a practical and widely adopted approach for exploring such landscapes, and repeated independent runs were used to increase confidence in the robustness of the identified solution.

As a second optimization tool, the golden search (GS) method was selected. It was selected as a refinement tool since it can achieve a minimum with fewer iterations in comparison to PSO. GS, as a deterministic method, always finds the same results for a data set. Similar to PSO’s approach, it can search for the best results within a specified interval, with the limitation of solving unidimensional problems. For the scope of this study, the method was applied to each dimension at a time, with a loop of two iterations. Further iterations were not able to achieve better results. It is worth pointing out that this strategy to use two optimization tools significantly reduces computational requirements, since using only PSO to find the minimum error would take up to 2 days for each algorithm round.

Regarding the computational cost of the optimization routine, each evaluation of the objective function requires 35 Aspen Plus simulations, corresponding to the number of experimental data points. Each simulation call took approximately 0.5 s, resulting in an evaluation time of about 17.5 s per particle per iteration. All computations were performed on a workstation equipped with an Intel Core i5–12400 CPU (6 cores, 12 threads) and 32 GB of RAM.

### 2.7 Algorithm

A brief description of the developed algorithm and its scheme can be found in Figure 3. The algorithm begins by initializing each particle with 20 dimensions for RS1 or 9 dimensions for RS2, representing sets of parameters randomly sampled according to PSO’s stochastic nature. These parameters are then converted into specific values and fed into a simulator. Within the simulator, 30 simulation runs are conducted for each particle set, encompassing a combination of five different temperatures and six residency times. During these runs, the simulator calculates the molar and mass fractions of reagents and products based on the given parameters.

**Figure 3:**
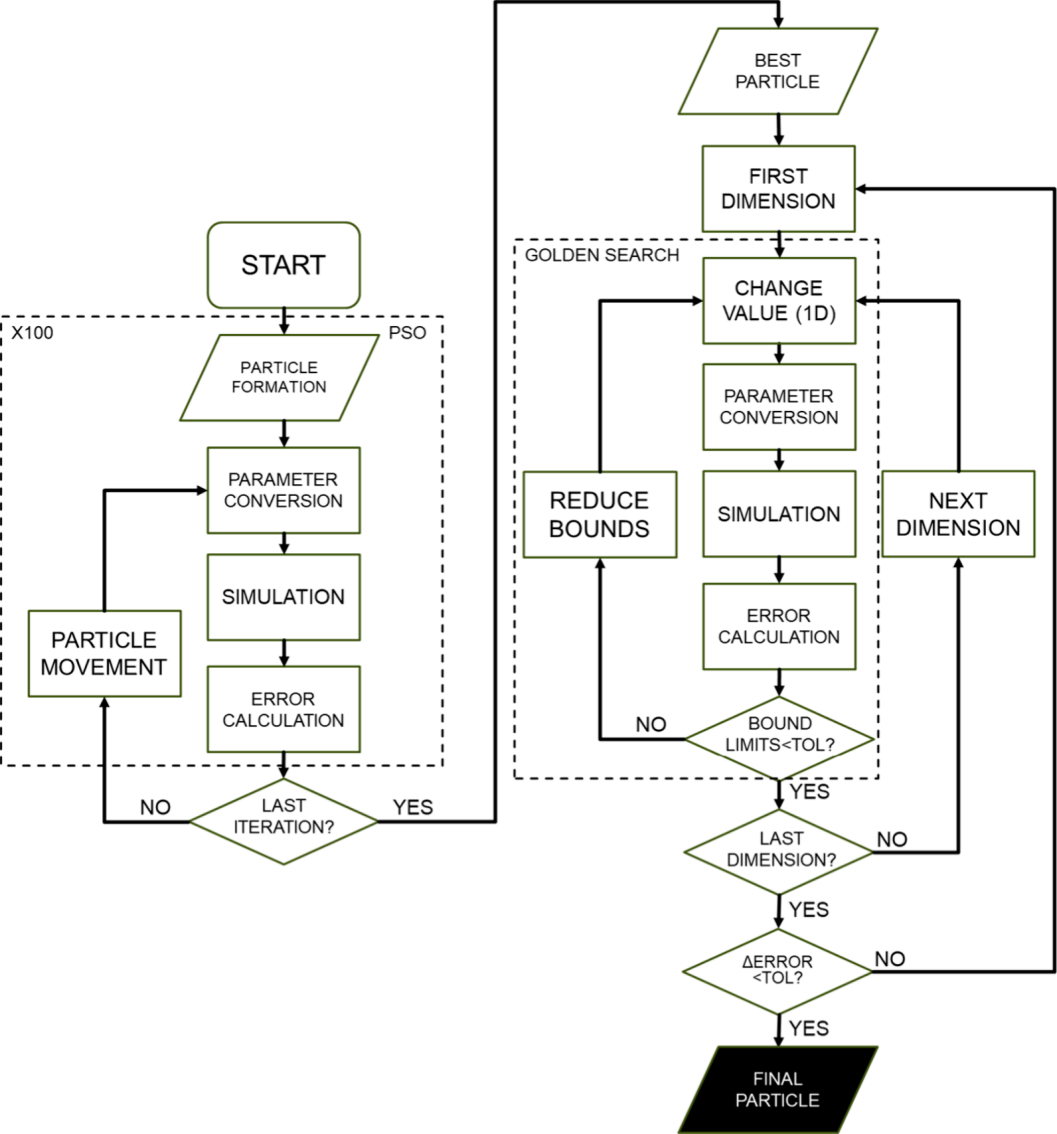
Algorithm scheme for kinetic parameter estimation.

Once simulated, the resulting fractions are compared against experimental values to compute the error metric using the objective function used in the current paper, i.e., the sum of squared errors (SSE), for each particle. This error evaluation process is repeated for all particles across 20 iterations of the PSO algorithm. Throughout these iterations, the particle with the parameters yielding the lowest error is identified as the best candidate for further refinement.

Following the PSO iterations, the particle identified with the minimum error undergoes refinement by using the golden section search tool. This method systematically narrows down the parameter space around the best particle’s parameter set, aiming to pinpoint the optimal values that minimize the error even further.

### 2.8 Confidence Interval

To evaluate the effect of each estimated kinetic parameter on the error calculated from the discrepancy between experimental and simulated values, one possible approach is to cause a disturbance to one parameter and recalculate the error. A confidence level must be defined to determine the error threshold beyond which errors are considered significant. In this work, the confidence level corresponding to one standard deviation (σ) is adopted.^11^

The *F*-statistic (also known as the *F*-test or Fisher’s *F*) is a statistical measure primarily used to compare the variances of two data sets and determine whether they are significantly different. The *F*-statistic is calculated as the ratio of two estimated variances:^12^

$$F=\frac{\mathrm{variances}\;\mathrm{between}\;\mathrm{groups}}{\mathrm{variances}\;\mathrm{inside}\;a\;\mathrm{group}}$$ 5

The value defined for *F* is known as the critical *F*-value. This value considers the degrees of freedom of the two groups analyzed as well as the established confidence level. The degrees of freedom for each group are defined as^13^

$$\mathrm{DF}=N_{\exp}N_{\mathrm{VAR}}-N_{\mathrm{PAR}}$$ 6

where DF represents the degrees of freedom, *N*_EXP_ is the number of experiments conducted, *N*_VAR_ is the number of measured variables or components, and *N*_PAR_ is the number of estimated parameters.

With the critical *F*-value, it is possible to obtain a set of parameter values that, when varied up or down, are considered statistically equivalent to the original result. In this work, these variations are defined as the margin of error for the parameters. The procedure to determine the margin of error involves introducing a positive perturbation to a parameter until the new error equals the minimum error multiplied by the critical *F*-value. This procedure is then repeated with a negative perturbation to the same parameter to obtain both bounds of the margin of error.

The degrees of freedom were computed as a single value encompassing all experiments, variables, and parameters rather than being determined individually for each reaction. This approach was chosen because the error calculation considers the entire reaction system, where variations in one reaction propagate through interconnected pathways, whether they are parallel or sequential.

## 3 Results and Discussion

### 3.1 Parameter Estimation

For RS1, at the final particle, which represents the optimal point, the error was 3563.41. In contrast, the best particle identified by PSO yielded an error of 4314.87. This optimal point reflects an average difference of 4.63% between the experimental and simulated values. Figure 3 displays the experimental and simulated conversions across the temperature range of stearic acid (SA) and palmitic acid (PA), while Figure 4 illustrates the profile of fatty acids and products over time at 613 K.

**Figure 4:**
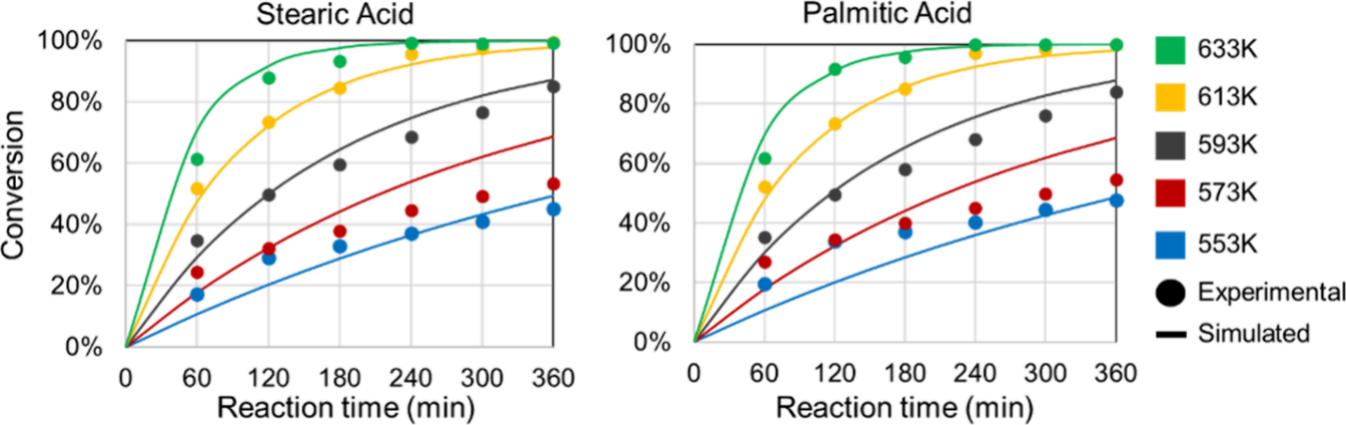
Time-dependent fatty acid conversion profiles vs temperatures at RS1.

The coefficient of determination (*R* ^2^) for SA conversion across the studied temperatures reached 0.945, while for PA conversion, it was 0.927, indicating a strong correlation between experimental and simulated values. Figure 3 illustrates that this correlation improves at higher temperatures. In Figure 4, *R* ^2^ values for SA products and PA products were notably high, at 0.995 and 0.989 respectively. Regarding product correlations with temperature variation, SA products showed an *R* ^2^ of 0.947, whereas PA products exhibited a slightly lower *R* ^2^ of 0.846. The overall correlation coefficient of 0.981 indicates a strong correlation between the proposed model and the experimental data. In Table 3, the obtained kinetic parameters are displayed.

**Table 3:** Kinetic Values Obtained by Optimization for RS1

reaction	*A*_0_ (kmol/kg_cat_ s)	*E*_*ai*_ (kJ/mol)	α
FA_2*k*_ → ALD_2*k*_	2.10 × 10^–6^	199.1	0.00
FA_2*k*_ → C_2*k*–1_	1.15 × 10^–5^	65.9	0.07
ALD_2*k*_ → ALC_2*k*_	5.97 × 10^–2^	149.5	0.10
ALC_2*k*_ → ALD_2*k*_	2.20 × 10^–5^	209.9	0.09
ALD_2*k*_ → C_2*k*–1_	2.08 × 10^–4^	209.7	0.11
ALC_2*k*_ → C_2*k*_	1.20 × 10^–5^	158.5	0.43

Particularly noteworthy are reactions ALD_2*k*_ → ALC_2*k*_ and ALD_2*k*_ → C_2*k*–1_, which feature higher *A*_0_ values compared to those of other reactions. This adjustment reflects the minimization tendency for aldehyde formation, a molecule that was absent in the experimental data. Furthermore, the alcohol to aldehyde reaction (ALC_2*k*_ → ALD_2*k*_) has a considerably high value for *A*_0_. This outcome seeks to mirror experimental data showing higher concentrations of alcohol compared with even-numbered *n*-alkanes. The α factor highlights a distinctive high value for reaction ALC_2*k*_ → C_2*k*_, underscoring the pronounced variation in even-numbered *n*-alkane production between stearic acid and palmitic acid reactions.

Overall, the production of odd-numbered *n*-alkanes was predominant compared to that of even-numbered ones. This outcome is driven by the catalyst selection, which employs nickel as the active agent, enhancing decarbonylation and decarboxylation reactions.^10^

Additionally, the adsorption constant (*K*_ADS_) was 1.80 m^3^/mol for stearic acid (SA) and 2.77 m^3^/mol for palmitic acid (PA). These values are significantly higher than those reported by Arora et al.,^8^ despite being based on a similar reaction scheme. This discrepancy may stem from differences in catalyst composition or the use of a fatty acid mixture as feedstock in this study, which can influence adsorption dynamics and reaction behavior.

Furthermore, the family activation energy (*E*_*ai*_) has higher values for reactions ALD_2*k*_ → C_2*k*–1_, ALC_2*k*_ → ALD_2_ *k*, and FA_2*k*_ → ALD_2*k*_, implying that their reaction rates would be more susceptible to temperature variations. In this subject, process energy optimizations could be important to balance reaction rates and energy consumption.

Optimal alfa factors were variable but especially low for reactions FA_2*k*_ → ALD_2*k*_, FA_2*k*_ → C_2*k*–1_, and ALC_2*k*_ → ALD_2*k*_. In these cases, there is a minor variation of specific activation energy within the family, revealing that the values for palmitic and stearic acid reaction routes would be very similar.^7^

For RS1, the complete optimization procedure required approximately 10.8 h per independent run, combining both the PSO stage and subsequent parameter refinement. Considering the five independent runs performed to ensure robustness, the total computational time was approximately 54.0 h.

For RS2, the optimized solution yielded an SSE of 2786.26, while the best particle identified by the PSO algorithm had an initial error of 3844.86. This corresponds to an average deviation of 4.10% between the simulated and experimental results. Compared with RS1, RS2 achieves a more accurate fit while utilizing a reduced reaction scheme, resulting in a 22% reduction in total squared error and an 11% decrease in average deviation. These improvements demonstrate the effectiveness of the model simplification.

Figure 5 presents the experimental and simulated conversion profiles of stearic acid (SA) and palmitic acid (PA) as a function of temperature, while Figure 6 illustrates the concentration profiles of fatty acids and their products over time at 613 K, and Figure 7 presents the product conversion profiles at the same temperature. The strong agreement between predicted and observed values confirms that RS2 successfully captures the system’s kinetic behavior.

**Figure 5:**
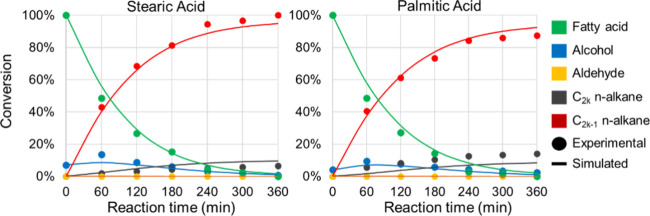
Time-dependent product conversion profiles at 613 K at RS1.

**Figure 6:**
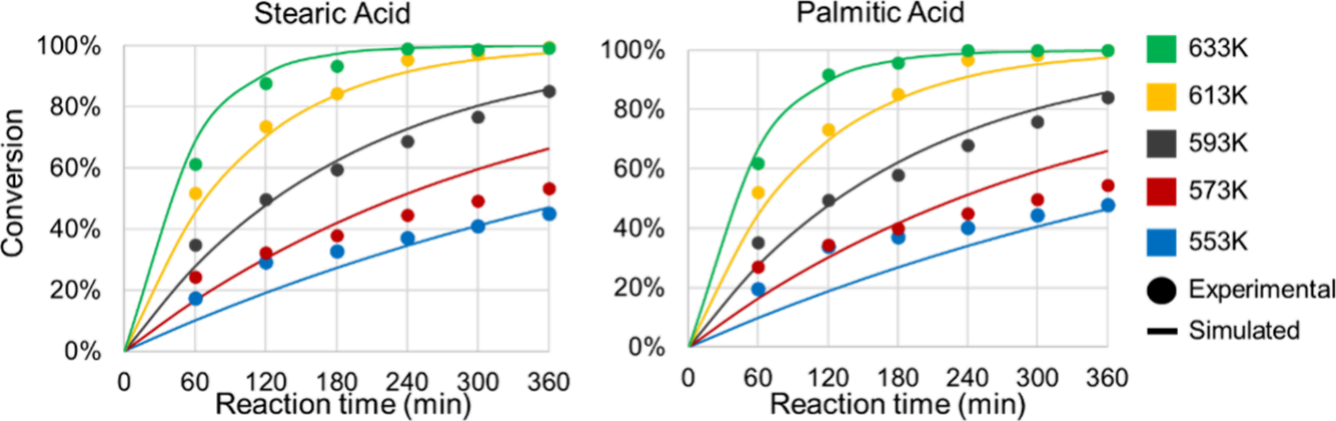
Time-dependent fatty acid conversion profiles vs temperatures at RS2.

**Figure 7:**
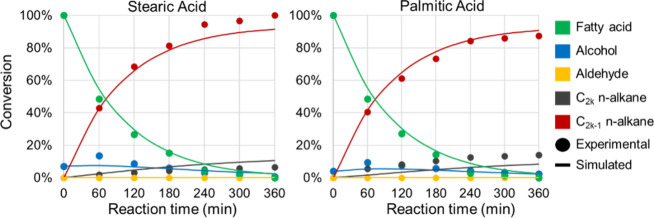
Time-dependent product conversion profiles at 613 K at RS2.

For RS2, the coefficient of determination (*R* ^2^) for stearic acid (SA) conversion across the studied temperature range reached 0.959, while palmitic acid (PA) conversion attained 0.942, both slightly higher than the corresponding values in RS1. In Figure 6, the *R* ^2^ values for SA and PA products were particularly high at 0.990 and 0.989, respectively, closely matching the excellent fits observed in RS1 (0.995 and 0.989).

When evaluating the correlation between product distribution and temperature variation, SA-derived products exhibited a *R* ^2^ of 0.980, while PA-derived products reached 0.933, both representing improvements over RS1. The overall correlation coefficient of 0.985—compared to 0.981 in RS1—demonstrates that the simplified reaction scheme (RS2) successfully preserves the predictive accuracy of the model while reducing its complexity. As noted by Kubička et al. (2013),^14^ the simultaneous occurrence of decarboxylation (DCx) and decarbonylation (DCn) is common, but the interplay between these reactions makes it challenging to precisely determine the origins of gaseous products. The kinetic parameters estimated for RS2 are summarized in Table 4.

**Table 4:** Kinetic Values Obtained by Optimization for RS2

reaction	*A*_0_ (kmol/kg_cat_ s)	*E*_*ai*_ (kJ/mol)	α
FA_2*k*_ → ALC_2*k*_	1.86 × 10^–6^	89.4	1.00
FA_2*k*_ → C_2*k*–1_	7.22 × 10^–6^	92.1	0.07
ALC_2*k*_ → C_2*k*_	2.03 × 10^–6^	154.7	0.02

These results highlight that reaction FA_2*k*_ → C_2*k*–1_ is the most favored pathway, while reaction ALC_2*k*_ → C_2*k*_ is the rate-limiting step, consistent with the predominance of odd *n*-alkanes in the product distribution. Reaction FA_2*k*_ → ALC_2*k*_ α values close to 1 indicate high sensitivity to structural differences between fatty acids. Comparing RS2 to RS1, where the α value was elevated for reaction ALC_2*k*_ → C_2*k*_, the different values regarding fatty acids being accounted in reaction FA_2*k*_ → ALC_2*k*_ were allowed due to the exclusion of the DCn route, letting alcohol only produce even-numbered *n*-alkanes.

For RS2, the complete optimization procedure required roughly 10.25 h per independent run. With five independent runs executed, the total computational cost reached approximately 51.23 h.

### 3.2 Confidence Interval

The estimated kinetic parameters were evaluated by using the confidence interval calculation algorithm. Considering RS1, Figure 8 presents the confidence interval for *A*_0_, while Figure 9 shows the confidence interval for *E*_*ai*_. The confidence interval for factor α is presented in Figure 10.

**Figure 8:**
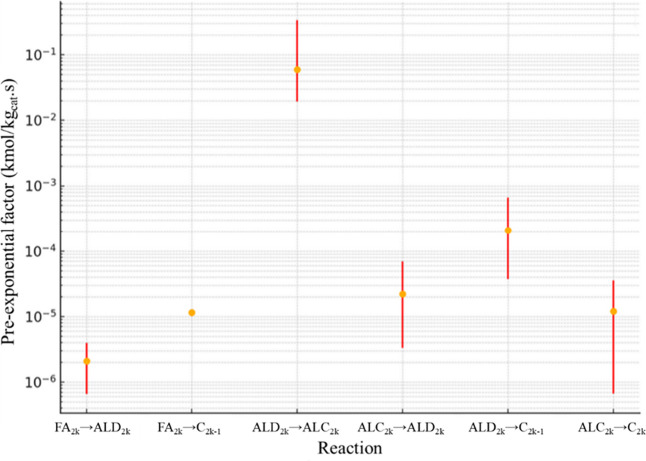
*A*_0_ confidence interval values (kmol/kg_cat_ s) for RS1.

**Figure 9:**
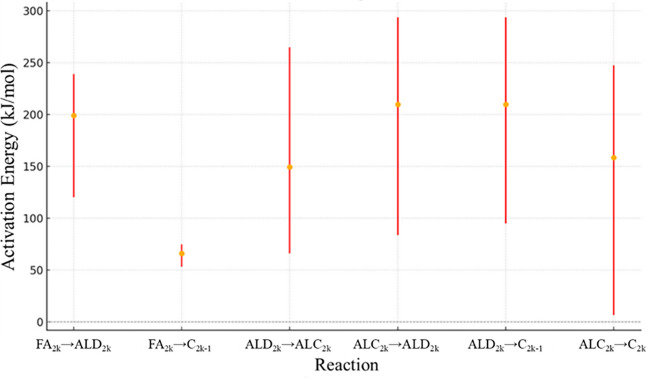
*E*_*ai*_ confidence interval values (kJ/mol) for RS1.

**Figure 10:**
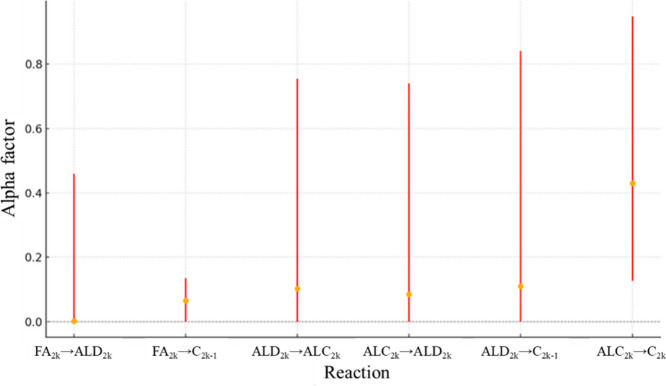
α confidence interval values for RS1.

The significance of the *A*_0_ value for reaction FA_2*k*_ → C_2*k*–1_, corresponding to the direct conversion of fatty acid to odd *n*-alkane, stands out due to the narrow margin of error between its upper and lower limits, imperceptible in Figure 8. This indicates that variations in this DCx pathway significantly impact the calculated error. When specifically comparing it to the parallel reaction forming odd *n*-alkanes, the pathway comprising reactions FA_2*k*_ → ALD_2*k*_ and ALD_2*k*_ → C_2*k*–1_ (DCn) exhibits a broader margin of error, resulting in less reliable outcomes.

It is observed that reaction ALD_2*k*_ → ALC_2*k*_ reaches the highest limit of the search region, indicating that higher rates for this reaction do not cause significant disturbances in the error.

For the reaction ALC_2*k*_ → C_2*k*_, the margin of error is notably shifted downward, suggesting that very low rates for this reaction also do not induce significant changes in the error.

The confidence interval spread was also evaluated by calculating the ratio between the upper and lower bounds for each reaction. For the most reliable pathway, FA_2*k*_ → C_2*k*–1_, this ratio was only 1.24, indicating narrow bounds and strong parameter identifiability. In contrast, for the least constrained step, ALC_2*k*_ → C_2*k*_, the ratio reached 53.41. The average ratio considering all reactions is 19.50. For reference, Arora and collaborators reported relatively narrow confidence intervals, varying from 1.29 to 2.84. It is also important to note that Arora et al.^8^ conducted a broader experimental exploration than Yenumala et al.,^7^ increasing the sensitivity of the kinetic fitting and contributing to the narrower confidence intervals reported in their work.

Reaction FA_2*k*_ → C_2*k*–1_ once again demonstrates the highest reliability regarding the activation energy estimate. Compared with the DCn pathway, the activation energy values indicate that DCx is favored by the chosen catalyst. Apart from reaction FA_2*k*_ → C_2*k*–1_, all other reactions exhibit a wide range of possible values, suggesting that variations in their parameters can be compensated by parallel reactions, minimizing their impact on the overall error calculation. For instance, the upper/lower confidence ratio for reaction FA_2*k*_ → C_2*k*–1_ is only 1.41, whereas for reaction ALC_2*k*_ → C_2*k*_ it reaches 37.72, with the average ratio considering all reactions being 8.62. For reference, Arora and collaborators^8^ report relatively narrow confidence intervals, varying from 1.43 to 16.62. These findings suggest that RS1 could be further optimized to enhance parameter reliability.

The confidence interval calculations for the α factors indicate that excluding them from the algorithm would not significantly impact the calculated error, except for reaction ALC_2*k*_ → C_2*k*_. Since the experimental data for the formation of even *n*-alkanes in the HDO pathway were the most discrepant between stearic acid and palmitic acid, this factor appears to be essential for capturing this difference in the simulations. Normalized confidence interval range (defined as the difference between upper and lower bounds over the explored search domain) for FA_2*k*_ → C_2*k*–1_ is 0.14 while ALD_2*k*_ → C_2*k*–1_ is 0.84, with an average value considering all reactions being 0.63.

The adsorption constants reached a lower search limit. The conversion of the particle fraction corresponding to the adsorption constants results in a value of 1 at the minimum search limit, which appears to be high considering that the authors modeled their kinetics by assuming this value to be 0.

The results for RS1 suggest that DCx is more reliable than DCn, since the latter presents broad confidence intervals for *A*_0_, *E*_*ai*_, and α, whereas DCx shows narrow bounds and therefore stronger statistical significance. Moreover, because gaseous products were not quantified experimentally, the CO/CO_2_ ratio could not be assessed, limiting the ability to distinguish DCn from DCx. Therefore, DCx alone is sufficient to explain the formation of odd-carbon hydrocarbons, making DCn redundant within the kinetic framework.

Removing DCn from the mechanism calls into question the need to maintain reversibility in the ALD_2*k*_ → ALC_2*k*_ step, especially considering that no aldehyde was detected experimentally. In addition, the fitted kinetic parameters indicate that ALD_2*k*_ → ALC_2*k*_ occurs much faster than the reverse step, suggesting that this conversion is effectively irreversible under the conditions evaluated. Consequently, the reverse reaction ALC_2*k*_ → ALD_2*k*_ can be removed from the mechanism without a loss of descriptive capability.

With DCn excluded and the ALD_2*k*_ → ALC_2*k*_ step shown to be effectively irreversible, the remaining question is whether it is necessary to represent FA_2*k*_ → ALD_2*k*_ and ALD_2*k*_ → ALC_2*k*_ as two distinct reactions. The system does not provide enough information to independently resolve these two intermediate steps since aldehydes were not detected experimentally. Therefore, FA_2*k*_ → ALD_2*k*_ → ALC_2*k*_ can be treated as a single lumped deoxygenation step, expressed as FA_2*k*_ → ALC_2*k*_.

In addition to the reaction-pathway simplifications, the adsorption parameters for stearic and palmitic acids converged to the lower boundary of the search space without significantly affecting the objective function. This behavior indicates that surface coverage effects are not required to reproduce the experimental trends and that the model is insensitive to variations in the adsorption terms. As a consequence, the kinetic rate expression can be simplified from a Langmuir–Hinshelwood formulation to a power-law form, further reducing the model complexity while maintaining predictive performance.

In summary, RS1 overparametrizes the system and leads to nonidentifiable steps. By retaining only the kinetically relevant pathways (DCx and HDO), lumping FA_2*k*_ → ALD_2*k*_ → ALC_2*k*_, removing reversibility, and dropping adsorption terms, a simplified reaction scheme (RS2) was formulated.

For RS2, Figure 11 presents the confidence interval for *A*_0_, while Figure 12 shows the confidence interval for *E*_*ai*_. The confidence interval for factor α is presented in Figure 13.

**Figure 11:**
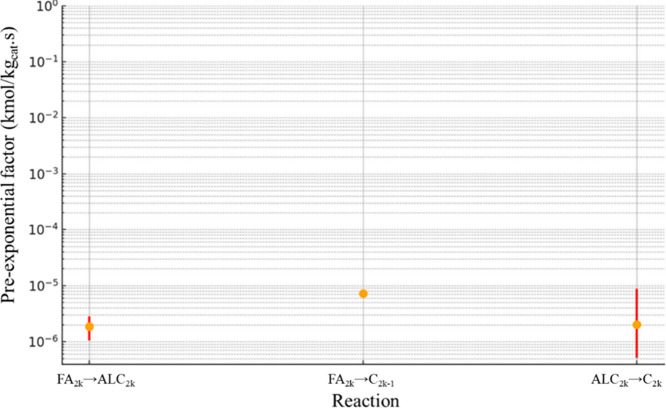
*A*_0_ confidence interval values (kmol/kg_cat_ s) for RS2.

**Figure 12:**
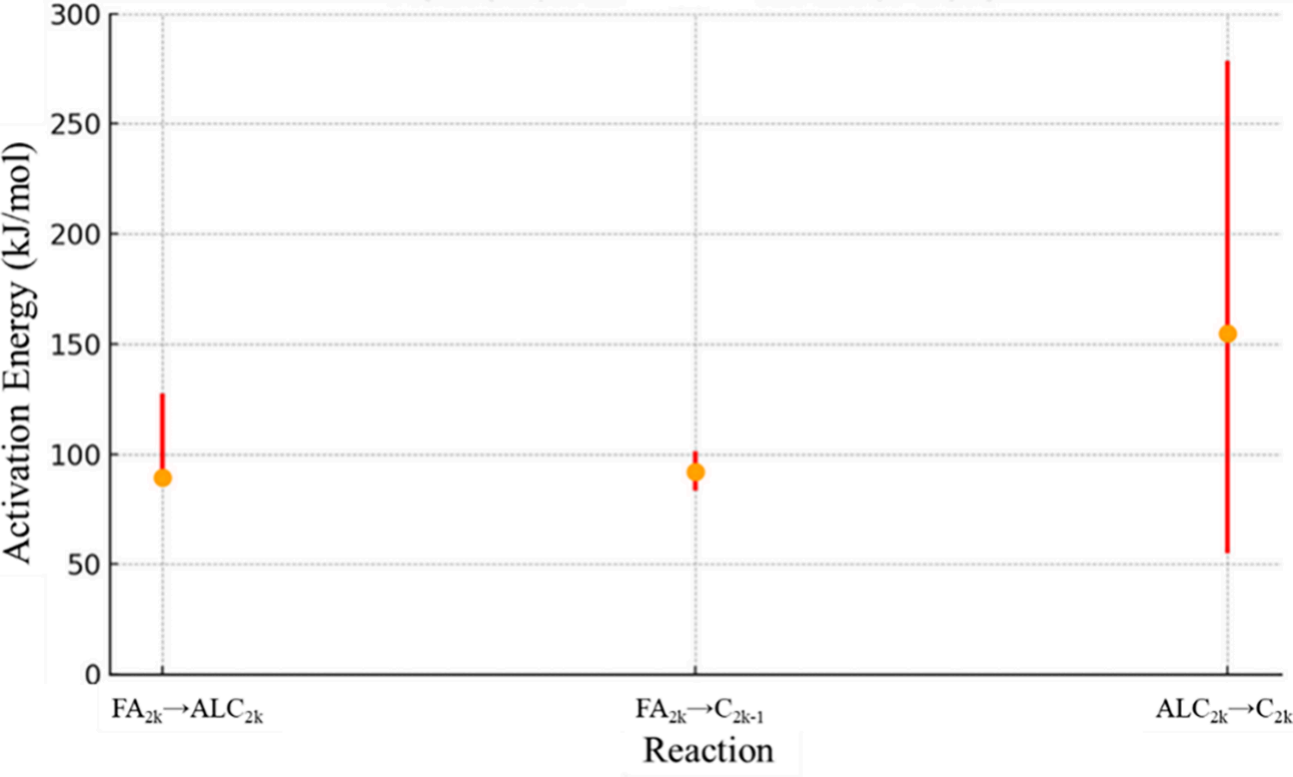
*E*_*ai*_ confidence interval values (kJ/mol) for RS2.

**Figure 13:**
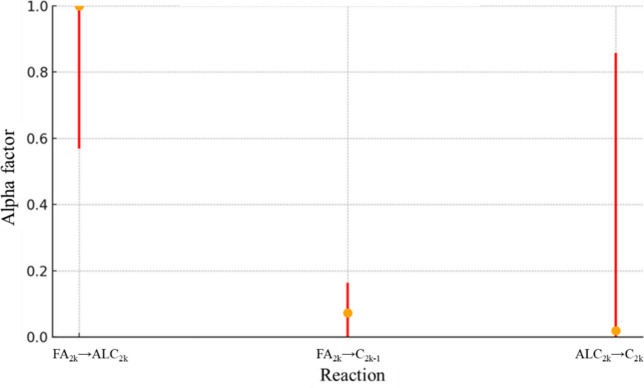
α confidence interval values for RS2.

It can be observed that RS2 exhibits greater reliability in estimating the pre-exponential factor compared to RS1, with reaction FA_2*k*_ → C_2*k*–1_ yielding the most consistent result, with an upper/lower ratio of 1.23, similar to the trend observed in RS1. Reaction ALC_2*k*_ → C_2*k*_, responsible for the production of even-numbered *n*-alkanes, demonstrates the lowest reaction rate among the three reactions. Consequently, its impact on global error is less significant, which justifies why its margin of error remains the largest among the reactions, with an upper/lower ratio of 16.97. The average ratio considering all reactions is 6.97. This highlights that while RS2 improves overall parameter estimation, the lower relevance of reaction ALC_2*k*_ → C_2*k*_ to the global system allows for greater variability in its parameter estimation.

Activation energy of reaction FA_2*k*_ → ALC_2*k*_ has decreased for RS2 in comparison to that for RS1. This change is not abnormal, considering that reaction FA_2*k*_ → ALC_2*k*_ is the merge of reaction FA_2*k*_ → ALD_2*k*_ and ALD_2*k*_ → ALC_2*k*_ from RS1. This, with all the described changes of reaction schemes, results in the possibility of obtaining different values for similar reactions. Additionally, the lower limit for reaction FA_2*k*_ → ALC_2*k*_ is the same as the optimized result. This is the result of Bells–Evans–Polanyi, eq 3, considering a high value for the α factor and a reaction enthalpy with negative values. To avoid negative values for activation energy, the lower bar for the reaction FA_2*k*_ → ALC_2*k*_ is suppressed. Values for reactions FA_2*k*_ → C_2*k*–1_ and ALC_2*k*_ → C_2*k*_ are similar to those found with RS1. Confidence interval ratios for activation energies in RS2 are considerably narrower than those in RS1, with a value of 1.22 for FA_2*k*_ → C_2*k*–1_, while the least constrained pathway (ALC_2*k*_ → C_2*k*_) still shows a broader interval with a ratio of 5.07. The average ratio considering all reactions is 2.57.

For reasons already discussed with the results from Table 4, the reaction FA_2*k*_ → ALC_2*k*_ plays a key role in differentiating the behavior of fatty acids. As a result, the reaction ALC_2*k*_ → C_2*k*_ inherits this differentiation effect, allowing it to distinguish between products derived from different fatty acids without requiring a significant alpha factor. This explains why reaction ALC_2*k*_ → C_2*k*_ shows low alpha values yet still reflects structural differences in its products. However, due to its lower contribution to overall product formation, reaction ALC_2*k*_ → C_2*k*_ remains the least reliable in terms of parameter estimation as its impact on the global error is minimal. Normalized confidence interval range for FA_2*k*_ → C_2*k*–1_ is 0.16 while ALC_2*k*_ → C_2*k*_ is 0.86, with an average value considering all reactions being 0.48.

## 4 Conclusions

In the present paper, a successfully conducted study estimated kinetic parameters for stearic acid and palmitic acid hydrodeoxygenation for green fuel production, using the linear free energy relationships (LFERs) approach, demonstrating a strong correlation between experimental and simulated values. Its application can be extended to complex feedstocks such as soybean oil, enabling kinetic parameter estimation while maintaining a manageable number of variables.

In exploring the multidimensional search space, an optimization PSO-based algorithm was developed to solve the problem and proved to be very effective. Following PSO’s identification of the particle with the lowest error, the golden section method was employed as a local optimizer, leading to a notable improvement in the error reduction.

The results obtained for RS1 and RS2, from applying the confidence interval, demonstrate that certain production pathways for *n*-alkanes from fatty acids are more significant in replicating the experimental data, such as reaction FA_2*k*_ → C_2*k*–1_, which represents the decarboxylation (DCx) pathway. This is true both because odd *n*-alkanes are the main product in Yenumala et al.’s^7^ experiments, increasing importance in the DCx pathway, and for catalyst features that facilitate the DCx pathway.

By reducing the complexity, RS2 achieved better results in terms of global error. Although the improvement in *R* ^2^ from RS1 to RS2 appears marginal, the total squared error was reduced by 22%, and the average deviation decreased by 11%. This improvement was achieved by using a simpler reaction scheme, which increases model parsimony and parameter identifiability. Results for RS2 were also better for the reliability of kinetic parameters, since the average confidence interval width for *A*_0_ decreased from 19.50 to 6.97, and for *E*_*ai*_ from 8.62 to 2.57, representing a strong reduction in parameter dispersion. Compared to Arora and collaborators,^8^ RS2 performs slightly worse regarding the confidence interval width. However, the feed in this study is more complex (mixed fatty acids), and the use of the α factor introduces extra variability, which explains the broader ranges. This highlights the advantage of optimizing a lower-dimensional parameter space, as performed in RS2, which facilitates convergence toward more accurate solutions. These findings suggest that, although RS1 was designed based on theoretical reaction pathways, the simplified approach adopted in RS2, where each experimental component is accounted for within a single reaction (whether as a reactant or a product), proved to be more effective in capturing the system’s behavior. This demonstrates that reducing the number of variables can enhance the model performance by minimizing uncertainties and improving parameter estimation accuracy.

These important findings can be integrated with additional kinetic data from diverse catalysts for a technical and economic evaluation of a green fuel production facility using triglycerides. Additionally, the data generated by optimization tools can be utilized to construct neural networks, which expedites the data gathering process.
